# Treatment Response of Deferiprone in Infratentorial Superficial Siderosis: a Systematic Review

**DOI:** 10.1007/s12311-020-01222-7

**Published:** 2021-01-06

**Authors:** Andreas Flores Martin, Priya Shanmugarajah, Nigel Hoggard, Marios Hadjivassiliou

**Affiliations:** 1 Academic Department of Neurosciences, Royal Hallamshire Hospital and University of Sheffield, Sheffield, UK; 2 Department of Infection, Immunity and Cardiovascular Disease, University of Sheffield, Sheffield, UK

**Keywords:** Superficial siderosis, Deferiprone, Ataxia, Infratentorial

## Abstract

Superficial siderosis describes haemosiderin deposition on the surface of the brain. When present on infratentorial structures, it can cause ataxia, sensorineural hearing loss and pyramidal signs. There is no proven treatment and patients experience slow progression of symptoms. Iron-chelating agents have been suggested as a therapeutic option and deferiprone is suited as it crosses the blood-brain barrier. However, deferiprone is reported to have a 1–2% risk of agranulocytosis. We performed a systematic review on treatment of infratentorial superficial siderosis with deferiprone based on PRISMA guidelines. Studies were included if in English or an English language translation was available, were about human subjects and referred to patients with ataxia. Studies were excluded if they did not possess an English translation, included animal studies or did not have ataxia. Studies were excluded if they discussed cerebral amyloid angiopathy or siderosis of other regions. Eleven papers were included. We identified 69 patients. Seventeen patients (25%) discontinued the drug. The most encountered adverse effect was anaemia (21.7%). Neutropaenia was observed in 8.7% and agranulocytosis in 5.8% of patients. Clinically, response varied, and stability or improvement was seen across neurological domains in 6 studies while 5 showed a mixed response. On imaging, 13 (28.9%) patients improved, 24 (53.3%) stabilised and 8 (17.8%) deteriorated. A prospective international centralised register of patients should be developed to inform the design and conduct of a multicentre, placebo-controlled, randomised clinical trial to evaluate the efficacy of deferiprone. The evidence from this systematic review is that deferiprone is a promising intervention.

## Introduction

Superficial siderosis is a rare disease entity that describes the deposition of iron-containing compounds, usually derived from blood breakdown products in the central nervous system. Originally described in 1908 based on pathological studies [1], the diagnosis was made at autopsy [2]. Due to advances in neuroimaging modalities which have a high sensitivity for haemosiderin, superficial siderosis can be identified *in vivo* on blood-sensitive magnetic resonance imaging (MRI) sequences such as susceptibility-weighted images and T2*-gradient recalled echo through recognising a curvilinear low signal intensity pattern [3].

Infratentorial superficial siderosis (iSS) is classified as haemosiderin deposition on the surface of at least two regions of the brain: cerebellum, brainstem, cranio-cervical junction, or spinal cord [1]. It is hypothesised to occur due to a dural tear which causes chronic persistent extravasation of blood into the subarachnoid space. This may be due to a variety of underlying aetiologies such as neurosurgery, trauma and tumours [4].

Haemosiderin deposition occurs due to the physiological process of iron sequestration. It is a protective mechanism which breaks down haemoglobin into haem, globin, ferritin, bilirubin, biliverdin and free iron. This complex process removes the toxic free iron and converts this into the less harmful haemosiderin which is deposited on the surface of the brain, causing loss of neurons and gliosis [5]. It is understood that chronic exposure to haemosiderin may damage the granule cells and purkinje cells [2, 6].

The process of haemoglobin neurotoxicity has been reported *in vitro* and *in vivo* studies [7, 8]. The mechanism is hypothesised to be multifactorial due to four main processes: oxidation, inflammation, nitric acid scavenging and oedema [9]. This damage is not reversible, and treatment therefore aims to halt further injury.

Clinically, this is a progressive and debilitating condition. Patients almost invariably present with a characteristic triad of progressive sensorineural hearing loss (95%), cerebellar ataxia (88%) and pyramidal signs (76%). Other signs and symptoms have also been described and commonly include headaches (37%), urinary problems (24%) and anosomia (17%), amongst others [10].

The need for a more standardised investigation and management approach has been proposed [4]. Patients presenting should be investigated thoroughly with brain and whole spine MR imaging or computerised tomography (CT) myelography to identify a possible source of bleeding. It is worth noting, however, that the chances of finding a source of bleeding are extremely small. If such a source is found, this can be surgically repaired to stop future leakage of blood and further haemosiderin build-up. Alternatively, iron-chelating therapy may be trialled.

There are presently no treatment options licensed for use. Iron-chelating agents have been identified as a promising treatment option. However, this is only licensed to prevent chronic iron overload in patients with thalassaemia. Deferiprone is uniquely suited for this purpose as it is lipid soluble and crosses the blood-brain barrier, targeting the central nervous system and reducing the chronic build-up of haemosiderin, thereby stopping progression [11]. Despite this, to date there has been no conclusive evidence that treatment alters the disease either clinically or radiologically [4]. Moreover, there have been concerns raised about the safety profile of deferiprone as it may cause life threatening adverse effects such as agranulocytosis. This was first reported by Huprikar et al. [12] in one patient after 4 months of therapy. It has been estimated to occur in up to 1–2% [13].

The aim of this study is to systematically review the available literature on the use of deferiprone in the treatment of infratentorial superficial siderosis by assessing the clinical and neuroimaging outcomes as well as the adverse effects experienced by patients.

## Methods

### Literature Search Strategy

A systematic literature search based on the PRISMA guidance was undertaken on PubMed, MEDLINE, PubMed Central and NCBI Bookshelf on 20 April 2020. The search involved using the Medical Subject Headings (MESH) terms described. Term A was “chelation therapy”, “chelating agents”, “pyridones” or “deferiprone” and Term B was “siderosis”, “hemosiderosis” or “superficial siderosis”. The inclusion and exclusion criteria detailed below were applied and the bibliography of each selected article was assessed for further studies which were not identified via the aforementioned strategy.

### Inclusion and Exclusion Criteria

To be included in the review, the following criteria had to be met:

1. Subjects were human
2. English language or English language translation available
3. Studies specifically referred to infratentorial/posterior fossa superficial siderosis
4. Studies referred to patients with superficial siderosis presenting with ataxia
5. Case reports, case series and trials

The exclusion criteria applied were as follows:

1. Studies with animal subjects
2. No English language translation available
3. Studies referring to only cortical superficial siderosis or cerebral amyloid angiopathy
4. Studies of patients without imaging who do not have ataxia
5. Studies not related to superficial siderosis of the central nervous system

## Results

**Fig. 1:**
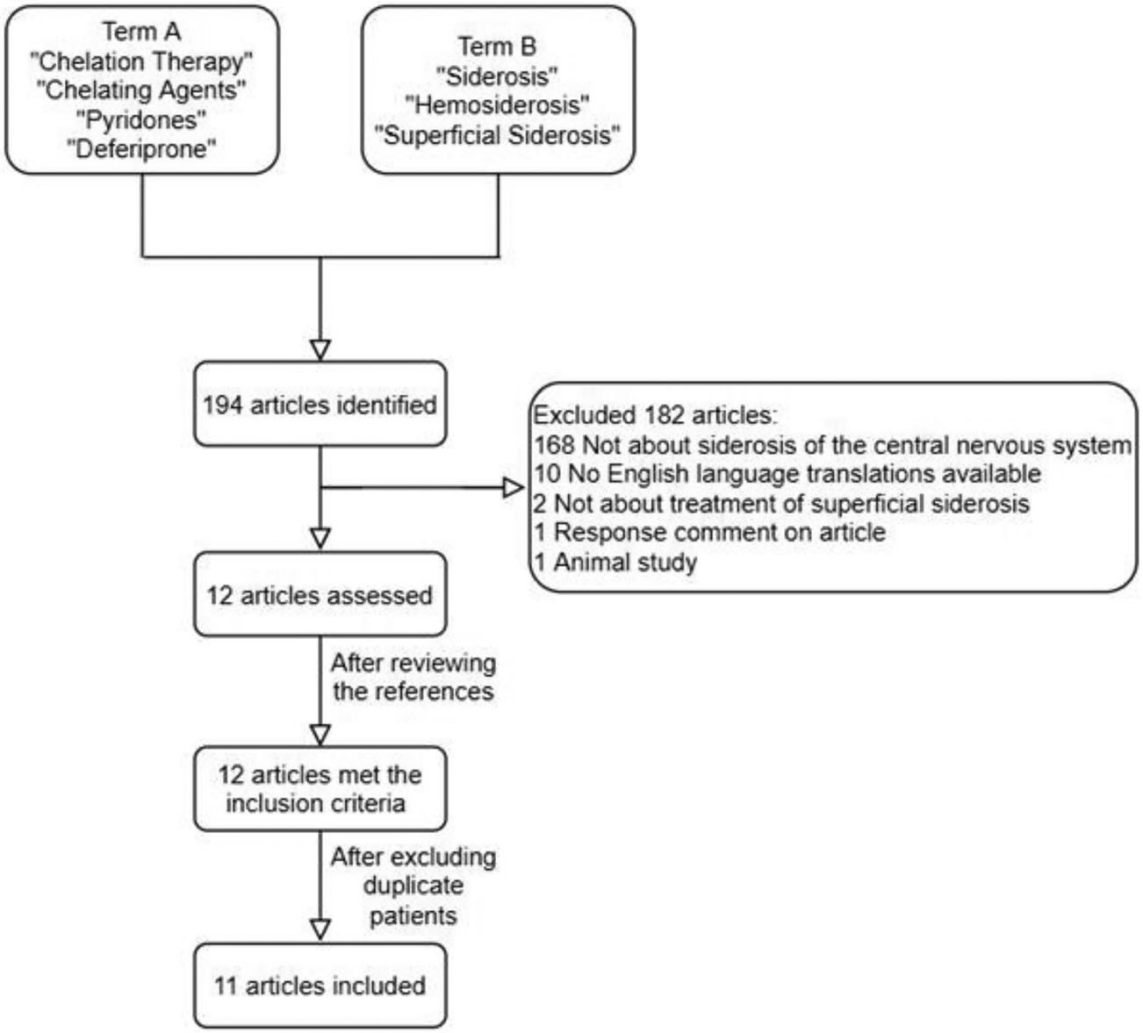
PRISMA chart illustrating study selection process

The search strategy resulted in the identification of 194 articles. The titles and abstracts were assessed against the eligibility criteria. Twelve articles met the inclusion criteria. One article was subsequently excluded as the same patient was reported in two separate studies [14, 15]. In total, 11 papers were used for this review. Figure 1 demonstrates the PRISMA diagram of study selection. Patient characteristics can be found in Table 1. The individual study characteristics can be found in Table 2.

**Table 1:** Patient characteristics

Number of iSS patients studied	Total number of iSS patients across all studies (*n*)	69
	Range of patients across all studies (*n*)	1–38
	Mean number of patients per study (*n*)	6.27
	Median number of patients per study (*n*)	1
Demographics	Male:female	14:9
	Mean age (years)	60.9
Imaging	Patients with pre-treatment MRI (*n*)	69
	Patients with post-treatment MRI (*n*)	43
Treatment	Mean treatment duration (months)	24.3
	Range treatment duration (months)	3–120
	Median treatment duration (months)	24
Tolerability	Patients who completed treatment (*n*)	57
	Patients who withdrew from treatment (*n*)	12

**Table 2:** Characteristics of included studies

Number of study	Author (year) reference	Study design	Patients (*n*)	Drug dose	Average disease duration (months)	Average treatment duration (months)	Outcome measures	Clinical change	Hearing	Ataxia	Radiological change	Adverse events	Results
1	Levy and Llinas [15]	Case report	1	15–30 mg/kg/day	N/A	38	Clinical and radiological change	Improved	Improved	Improved	Improved	Iron deficiency and fatigue	Positive
2	Levy and Llinas [16]	Interventional safety trial	10	30 mg/kg/day	144	3	Adverse effects	Mixed	Not documented	Not documented	Mixed	Iron deficiency and deranged liver function tests	Mixed
3	Cummins et al. [17]	Case report	1	1000 mg three times a day	84	12	Clinical and radiological change	Improved	Improved	Improved	Stable	Nil	Positive
4	Huprikar et al. [12]	Case report	1	1000 mg twice a day for 5 days a week	276	4	Clinical and radiological change	Stable or improved	Stable	Improved	Stable	Agranulocytosis	Mixed due to side effects
5	Schirinzi et al. [18]	Case report	1	30 mg/kg/day	60	3	Clinical and radiological change	Improved	N/A	Improved	Stable	Nil	Positive
6	Kuo et al. [19]	Case report	1	15 mg/kg/day	72	6	Clinical and radiological change	Improved	Improved	Improved	Improved	Nil	Positive
7	Derle [20]	Case report	1	30 mg/kg/day	N/A	9	Clinical and radiological change	Stable	Stable	Stable	N/A	Nil	Positive
8	Kessler et al. [21]	Longitudinal observational study	38	30 mg/kg/day for 5 days a week	102	24	Clinical and radiological change	Mixed	13 stable, 18 worse	2 improved, 8 stable, 21 worse	2 improved, 10 stable, 4 worse	10 fatigue, 5 heavy metal deficiency, 2 neutropaenia, 2 joint pain, 2 mouth sores	Mixed
9	Cossu et al. [22]	Case series	4	15 mg/kg twice a day	93	44.4	Clinical and radiological change	Stable or improved	Not documented	2 improved, 2 stable	4 improved	Nil	Positive
10	Levy [23]	Case report	1	1500 mg twice a day	24	120	Clinical and radiological change	Mixed	Stable	Worse	Improved	Nil	Mixed
11	Sammaraiee et al. [24]	Case series	10	10–30 mg/kg/day	31.8	27.6	Clinical and radiological change, adverse effects	6 stable, 4 worse	Not documented	Not documented	8 stable, 2 worse	4 iron deficiency, 3 agranulocytosis, 1 fatigue, 1 joint pain	Mixed

### Aetiology

The underlying aetiology for infratentorial superficial siderosis is variable and may be difficult to confidently identify. The largest available study by Kessler et al. [21] did not report all the aetiologies but noted that a dural tear was the commonest one while many remained unknown despite investigation. The most common source of bleeding was due to dural defects (11.5%). 6 patients (8.7%) had no identified source. Less commonly 7.2% occurred post-neurosurgery, 4.3% had a CNS tumour and 2.9% had a meningocoele. Rarer causes, each diagnosed in 1 patient, were reportedly seen due to subarachnoid haemorrhage, vertebral artery aneurysm, recurrent inner ear haemorrhage, road traffic accident and frontal subdural.

### Disease Duration

The data on the duration of illness prior to commencing treatment was not routinely reported. Of those studies which reported it, the duration of disease prior to therapy was between 6 months [24] and 276 months [12]. The average documented disease duration for positive outcomes was 77.3 months and for mixed outcomes was 115.6 months, raising the possibility of earlier intervention providing better outcomes for patients.

### Deferiprone

Deferiprone is currently licensed for the treatment of iron overload in patients with thalassaemia major in whom desferrioxamine is contraindicated or is inadequate. This iron-chelating agent was found to be safe, effective and well tolerated in multicentre safety and efficacy trials [25, 26]. It is prescribed at a dose of 75 mg/kg/day, given 3 times a day (rounded down to the nearest 250 mg tablet).

As deferiprone is the only agent that crosses the blood-brain barrier, this review evaluated its use in the treatment of superficial siderosis. The first case report on its use was published by Levy and Llinas [14] in 2011. Due to promising findings of improved clinical and neuroimaging features, further studies were performed.

In infratentorial superficial siderosis, there has been variation in the treatment dose and the optimal dose remains unclear. Levy and Llinas [14] commenced treatment at a dose of 30 mg/kg/day and subsequently reduced the dose to 15 mg/kg/day due to perceived side effects. This treatment regime has been used in multiple other cases including a pilot safety trial for the drug [14–16, 18, 20]. However, other treatment doses have been reported in literature including 30 mg/kg/day over 5 days [21], 15 mg/kg/day [19, 22], 10–30 mg/kg/day [24], 1000 mg twice a day for 5 days [12], 1500 mg twice a day [23] and 1000 mg three times a day [17]. These doses are lower than the therapeutic 75 mg/kg/day licensed use for thalassaemia.

The reported treatment duration varied considerably. In a case report, Levy [23] shares his experience of a single patient who was treated for 10 years with deferiprone. The average treatment duration was 24.3 months. However, the range of treatment duration was between 3 months and 120 months, with a median of 24 months.

These factors limit the interpretation of findings as higher doses may lead to increased adverse effects which are not experienced with lower doses [14]. This contrasts with the known dose and side effect profile of deferiprone when used for thalassaemia. Moreover, as different doses have been used across studies, there remains no optimal therapeutic dose available.

### Treatment Effect—Clinical Change

The first report that identified a positive clinical change with deferiprone was published in 2012 [15]. Following treatment with deferiprone for 38 months, the patient reported that the hearing loss and ataxia had resolved and at the time of publication the patient was asymptomatic. In a previous publication on the same patient, Levy and Llinas [14] reported that his hearing had stabilised while the ataxia had improved within 6–12 months. Clinically, 5 case reports identified improvement in ataxia following treatment [12, 15, 17, 18] while only 3 patients reported a subjective improvement in hearing [15, 17, 19]. In a pilot safety trial for the use of deferiprone, Levy and Llinas [14] were able to show that after 3 months, 4 (40%) clinically improved, 4 (40%) were stable and only 2 (20%) were worse. However, this trial was not designed to measure clinical outcomes and may have introduced bias as 8 (80%) patients self-reported the outcome.

The largest study by Kessler et al. [21] involved 38 patients who were followed up for 2 years, with 31 completing the clinical trial. This identified that 19 (63%) of their cohort reported a stable or improved clinical outcome in at least one domain (ataxia or hearing) with 40% showing a stability in their hearing and 30% a stable or improved coordination and walking. On the other hand, 11 (37%) identified a slow progression of their clinical condition and overall, the results were mixed as both positive and negative changes were seen in individual neurological domains across patients.

Similarly, after therapy with deferiprone for 10 years, Levy [23] reported that there was stabilisation of hearing and worsening of ataxia. Moreover, in a case series no clinical improvement was identified in 6 (60%) patients’ condition and 4 (40%) had deteriorated [24]. Therefore, these studies showed that deferiprone showed promise as a medication to stop or slow disease progression.

There were a number of limitations in these studies. One study was not designed to monitor the clinical outcomes [16], clinical change was not objectively assessed by a neurologist in 2 studies [12, 23] while Kessler et al. [21] reported both objective and subjective assessments [1, 6, 10, 13, 21]. As studies are largely case reports, these findings may not be generalisable. Furthermore, there had been many patients who had stopped taking treatment due to adverse effects or due to the cost of the drug [21, 24]. Lastly, outcome measures were inconsistent as no standardised reporting protocol had been used and changes were identified as overall difference as well as domains (ataxia and hearing impairment).

### Treatment Effect—Radiological Change

Out of the 45 patients who had repeated magnetic resonance imaging across 11 studies, 13 (28.9%) showed subjective radiological improvement, 24 (53.3%) showed no change and 8 (17.8%) showed a deterioration. One study did not report MRI findings after treatment [20]. In the largest study, Kessler et al. [21] report that only 16 (42.1%) of 38 patients completed the trial with an MRI.

Reporting of imaging modalities was found to be poor. MRI field strengths were only reported in 2 of the studies and one of these used both 1.5 T and 3 T on the same patient which may account for the lack of improvement as haemosiderin deposits that was not previously identified may now be seen [16, 22]. The included studies were noted to use different MRI sequences and protocols. Six studies were found to use blood-sensitive MRI sequences (susceptibility-weighted images and T2* gradient recalled echo) [15, 19, 21, 22, 24]. Three only used T2 sequences [12, 16, 17]. Three did not report imaging modalities [18, 20, 23].

Cossu et al. [22] reported that the MRI findings varied on 6 monthly follow-up and although unchanged at 6 months, by 12 months 2 patients were found to have a reduction in haemosiderin deposition and this improvement persisted up to 60 months. This raises the issue of whether patients in other studies have not been followed up for an appropriate length of time to appreciate the radiological changes.

This review is limited as not all patients had repeated MRI sequences after treatment. Only one of the studies used single blinded evaluation by the reporting radiologist [22]. There was also no standardised timescale for repeat neuroimaging follow-up. Moreover, at present, there is no validated quantitative method to identify the amount of haemosiderin deposition on MRI and the reporting seen in these studies is subjective and observer-dependent. Lastly, only 2 studies used multiple imaging assessors [22, 24]. Due to this, radiological changes must be interpreted with caution.

### Adverse Effects

Two observational studies designed to monitor side effects and the safety of deferiprone were available. Levy and Llinas [16] reported that patients who received the drug for 3 months were monitored with monthly blood tests. Iron deficiency anaemia developed in all patients and 3 developed abnormal liver function tests. Of note, Levy and Llinas [16] may have introduced a selection bias as he excluded patients who had previously experienced similar adverse effects. Similarly, Cossu et al. [22] reported that in his cohort, no adverse effects were present, and the drug was well tolerated.

Significant concerns regarding agranulocytosis were raised in a case report by Huprikar et al. [12]. A patient developed a total neutrophil count of 0 after 4 months of therapy and 3 months of unremarkable biochemical monitoring. This is the only documented case which required multiple prolonged intensive care unit admissions [12].

When taking into account all available studies, iron deficiency anaemia was the most common side effect in 15 (21.7%) patients [15, 16, 24]. This was closely followed by fatigue in 12 (17.4%). Other less commonly seen side effects included heavy metal deficiencies (7.2%), joint pain (4.3%), abnormal liver function tests (4.3%) and mouth sores (2.9%). Kessler et al. [21] report that fatigue appeared to be dose-dependent.

The more serious side effects of neutropaenia and agranulocytosis were present in 6 patients (8.7%) and 4 patients (5.8%) respectively. All 4 patients with an identified agranulocytosis were previously found to be neutropaenic clearly showing the importance of close monitoring for adverse events. Out of the 6 patients who developed a neutropaenia, 5 of them were re-challenged with deferiprone and in these 3 cases, agranulocytosis (defined as an absolute neutrophil count of 0.5 × 10^9^/L or lower), occurred [24]. Two patients did not progress despite re-challenging with treatment [21]. This is greater than the 1–2% risk of agranulocytosis previously reported [13]. Two patients benefited from treatment with granulocyte colony stimulating factor (GCSF) [12, 24].

The length of deferiprone therapy prior to neurotropaenia was not documented individually and cannot be confidently assessed. Time for neutrophil recovery for all patients varied between 0.33 and 3 months. However, this is partly confounded by the prescription of GCSF in 2 individuals [12, 24].

Onset of adverse effects after commencing treatment was not routinely documented in the studies. From the limited literature available, these side effects were not related to duration of treatment. While the identification through routine monitoring was detected as early as 90 days in one study [16], one patient presented with a neutropaenic sepsis after 30 months [24]. Similarly, one patient exhibited no side effects after 10 years [23].

The reported adverse effects were most commonly seen within the same studies. All patients who experienced heavy metal deficiencies and mouth sores were identified by Kessler et al. [21]. Similarly, in the pilot study, Levy and Llinas [16] reported all ten 10 patients experienced iron deficiency which made up 66% of the total reported cases. Deranged liver function tests were only seen in this study. It is important to note that the increase from 1 to 4 patients with agranulocytosis is due to one retrospective study rather than a common finding across all studies [24]. On the other hand, the 6 cases of neutropaenia were reported across three studies [12, 21, 24].

The adverse effects documented varied from the known reported side effect profile of deferiprone [27]. Most commonly, nausea, vomiting or abdominal pain were experienced by 33% of patients. None of the 69 patients in our review developed these adverse effects. Arthralgia was seen in 15% of patients with thalassaemia and 4.35% of patients with siderosis. This may be due to the lower dose used. Neutropaenia was present in 9.1% and 8.7% respectively. On the other hand, fatigue was only witnessed in superficial siderosis.

These studies are also limited as monitoring protocols for adverse effects were either not reported or found to be inconsistent across the available literature. When used in thalassaemia, the European Medicines Agency advises weekly full blood counts for the first year of treatment and may be extended thereafter on a patient to patient basis [28]. Of the reported protocols, only five studies reported commencing with weekly full blood counts with decreasing regularity after this period [12, 15, 16, 22, 24]. One study reported monthly bloods tests [21]. Five studies did not report the frequency of biochemical monitoring [17–20, 23]. Due to this, adverse effects may be identified and acted upon late.

### Tolerability

Of the reported cases, 12 (17.4%) of patients discontinued treatment. Of these, 5 (7.2%) were due to neutropaenia, 4 (5.8%) due to cost, 1 (1.5%) due to agranulocytosis, 1 (1.5%) due to fatigue and 1 (1.5%) due to complications related to another medical illness. However, in some cases, treatment was discontinued out of caution due to reduced neutrophil count and re-challenged despite neutrophils returning to baseline [21].

This study has several important factors to highlight. A key strength of this study was that the protocol was established prior to commencing the review process and was registered prospectively on PROSPERO. As this is a review of the available literature, the risks of a publication bias should be acknowledged. There are only three trials of 10 or more patients, and the results may be skewed in favour of these results over smaller case studies and series. Furthermore, as this is a retrospective study, the data collected relies on the published material which may be incomplete. One should note that this was a heterogenous population with varying aetiologies, disease and treatment durations which precludes a meta-analysis.

## Conclusions

This review identified a rather limited literature on deferiprone in the treatment of infratentorial superficial siderosis. The methodology used in each study was different and reporting of outcome measures found to be inconsistent. There was only one large-scale observational study thus interpretation of results should be made with caution and drawing conclusions from the cohort as a whole remains limited.

Furthermore, it is clear that evidence for clinical and radiological change can take a few months to years to develop and it is not known whether radiological severity is correlated with clinical severity. As these studies are largely short-term 6-12-month case reports, the outcomes are more likely to be negative. Similarly, as disease duration is often not reported for individual patients, the timing between onset of symptoms and treatment could not be identified. This raises the question of whether early intervention would be more beneficial to patients.

Overall, deferiprone appeared to be well tolerated and discontinuation was due to mainly limited experience as well as other unrelated conditions. However, the results showed that in some cases deferiprone can be successfully used for the treatment of infratentorial superficial siderosis to achieve clinical and radiological stability and in some cases improvement. This should be coupled with close monitoring of the patient’s full blood count, haematinics and trace minerals to ensure adverse effects are identified and acted upon. Due to the rarity of the condition and treatment, we also advise ensuring patients are educated on the possible effects to identify and guide their treating physicians to the important but lesser known adverse effects.

Ideally, large-scale, multicentre, placebo-controlled, randomised studies should be performed using a standardised protocol which includes treatment dose, duration of follow-up and outcome measures (audiometry, scale for the assessment and rating of ataxia (SARA) scores and adverse effect monitoring) to provide further evidence of the role of deferiprone in the treatment of infratentorial superficial siderosis. There is also a need for further detailed stratification of patients to accurately identify factors which alter patient outcomes such as underlying cause of superficial siderosis, disease duration and severity. Moreover, a prospective international centralised register of patients should be developed which could inform the design and conduct of a multicentre, placebo-controlled, randomised clinical trial to evaluate the efficacy of deferiprone.

## Data Availability

PROSPERO 2019 CRD42019142336 Available from: https://www.crd.york.ac.uk/prospero/display_record.php?ID=CRD42019142336
